# Inductive cum targeted yield model-based integrated fertilizer prescription for sweet corn (*Zea mays* L. *Saccharata*) on *Alfisols* of Southern India

**DOI:** 10.1371/journal.pone.0307168

**Published:** 2024-08-26

**Authors:** Krishna Murthy Rangaiah, Bhavya Nagaraju, Govinda Kasturappa, Annappa Nevatoor Nagendrachari, Basavaraja Pujari Kadappa, Uday Kumar Sugaturu Narayanaswamy, Mohamed Saqeebulla Hussain Sab, Gangamrutha Godekere Veerabadraiah, Sanjay Srivastava, Pradip Dey

**Affiliations:** 1 All India Coordinated Research Project on Soil Test Crop Response, University of Agricultural Sciences, Bangalore, Karnataka, India; 2 All India Coordinated Research Project on Soil Test Crop Response, ICAR-Indian Institute of Soil Science, Bhopal, Madhya Pradesh, India; 3 Indian Council of Agricultural Research-Agricultural Technology Application Research Institute, Kolkata, West Bengal, India; University of Minnesota, UNITED STATES OF AMERICA

## Abstract

Striking the right nutrient balance is essential for sustainable farming and ecosystem health. In this regard, field experiments were conducted in three phases *viz*., fertility gradient experiment, main experiment and validation experiment through a soil test crop response approach to develop and validate fertilizer prescription equations for sweet corn in comparison with general recommended dose and soil fertility rating approach. The soil data, fresh cob yield, and NPK uptake were used for establishing four important basic parameters, *viz*., nutrient requirement (kg t^-1^), contribution of nutrients from fertilizers, soil, and organic manure. The results revealed that nutrients required to produce one tonne of fresh cob yield (NR) were 5.85 kg, 0.87 kg and 4.31kg for N, P and K, respectively under the STCR NPK alone approach and 6.07 kg, 0.92 kg and 4.33 kg for N, P and K, respectively under STCR NPK+FYM approach. In the validation experiment, STCR NPK+FYM approach for the targeted yield of 25 t ha^−1^ recorded higher fresh cob yield (23.38 t ha^−1^) and dry stover yield (35.07 t ha^−1^) which were significantly higher compared to general recommended dose and soil fertility rating approach. The developed STCR equations for the aforesaid crop are valid as the percent deviation of cob yield from the targeted yield was within ±10%. Similarly, highest nutrient use efficiency was achieved with the STCR approach, specifically when targeting a lower yield through an NPK+FYM mode. Thus, implementation of the STCR approach of fertilizer prescription, with or without FYM, at targeted yields of 25 and 22 t ha^−1^, not only surpassed the effects of the other fertilizer recommendation approach in terms of cob yields, but also increased NPK uptake, improved nutrient use efficiency and greater economic returns.

## Introduction

Restoring soil nutrients and fixing nutritional imbalances using fertilizers boosts agricultural yields, plant health, and food security. However, it’s important to use fertilizers wisely to prevent environmental problems and optimize crop production [1]. Fertilizers are necessary to ensure that plants receive the essential nutrients for growth. Soil testing helps farmers to detect nutrient deficiencies and enables precise fertilizer recommendations. Among various approaches to fertilizer recommendation, the soil test crop response approach aims at boosting crop yields, reduces waste and supports sustainable agriculture. It relies on understanding soil traits, nutrient availability, crop responses and optimizing fertilizer use. This methodology serves to safeguard the environment by mitigating the excessive application of fertilizers and diminishing the potential hazards associated with runoff and leaching. Over an extended period, this practice reduces the expenses associated with fertilizer use, confirms to principles of sustainability, and guarantees the availability of food. The utilization of this practice in contemporary agriculture serves as a fundamental pillar, yielding advantages for farmers, the environment, and society at large.

The Soil Test Crop Response (STCR) approach provides fertilizer recommendations considering the nutrient composition of the soil. This boosts crop yields efficiently, particularly with high-yielding crop varieties. The combination of escalating fertilizer expenses and restricted availability frequently results in inadequate and disproportionate fertilization practices, so impeding the crop yield potential and compromising soil quality. Consequently, farmers experience economic losses as a result of this situation. In the process of setting fertilizer quantities, one should consider multiple factors such as crop response, nutritional needs, local nutrient source availability, and their immediate and future impact. Sweet corn (*Zea mays var*. *saccharata*) has gained popularity across the world owing to its sweet, creamy, tender, crispy and almost shell-less kernels [1]. Sweet corn is one of the most popular vegetables in the USA, Canada and Australia. It is gaining popularity in India and other Asian countries [2]. The green cobs are harvested at the dough stage and the kernels contain 18–20% carbohydrates, 5–6% free sugar, 2.1–4.5% proteins and 70% water. In India, sweet corn was grown on 9.22 million hectares, yielding an average of 2.92 t ha^−1^ [3]. This equates to a total output of 26.88 million tons. In Karnataka, poor sweet corn production relative to national productivity may be attributable to less use of advanced crop management technology, such as planting density, nutrition, plant protection, irrigation, etc. Crop yield depends on plant nutrition and other factors [4]. Sweet corn removes a lot of nutrients from the soil, therefore balanced fertilization may affect its output. Imbalanced use of chemical fertilizers is a common practice adopted by majority of farmers. Extensive use of chemical fertilizers causes problems with groundwater and environmental pollution through leaching, volatilization, denitrification, and wastage of nutrients through costly fertilization [5]. To attain maximum productivity for the crop and to maintain the fertility of the soil, balanced nutrition has to be given [6].

In this regard, the STCR methodology proves valuable for estimating crop-specific fertilizer needs. The correlation between nitrogen, phosphorus, and potassium (NPK) requirements and the targeted yield (TY) is linear and depends upon the prevailing soil test values (STVs). The STCR approach suggests fertilizer levels for a crop after establishing a strong link between soil test values (STVs), added fertilizer nutrients, and crop response in a soil type. This recommendation is derived either through fertilizer adjustment equations or targeted yield equations [7]. Thus, precise fertilizer recommendations can be made using this approach, as it involves data from soil and plant analysis [7], This technique also incorporates fertilizer, soil and organic manure nutrient contributions [8]. Recent sweet corn varieties benefit from nutrients, and sustained production requires better nutrition management. Thus, this study was conducted with objectives to a) develop fertilizer equations with sole NPK fertilizers and NPK + FYM using STCR methodology, b) validate these equations for targeted yield, and c) compare STCR treatments to current nutrient management strategies for fresh cob and dry stover yield, economics, nutrient uptake, and nutrient use efficiency.

## Material and methods

### Experimental site

**Fig 1:**
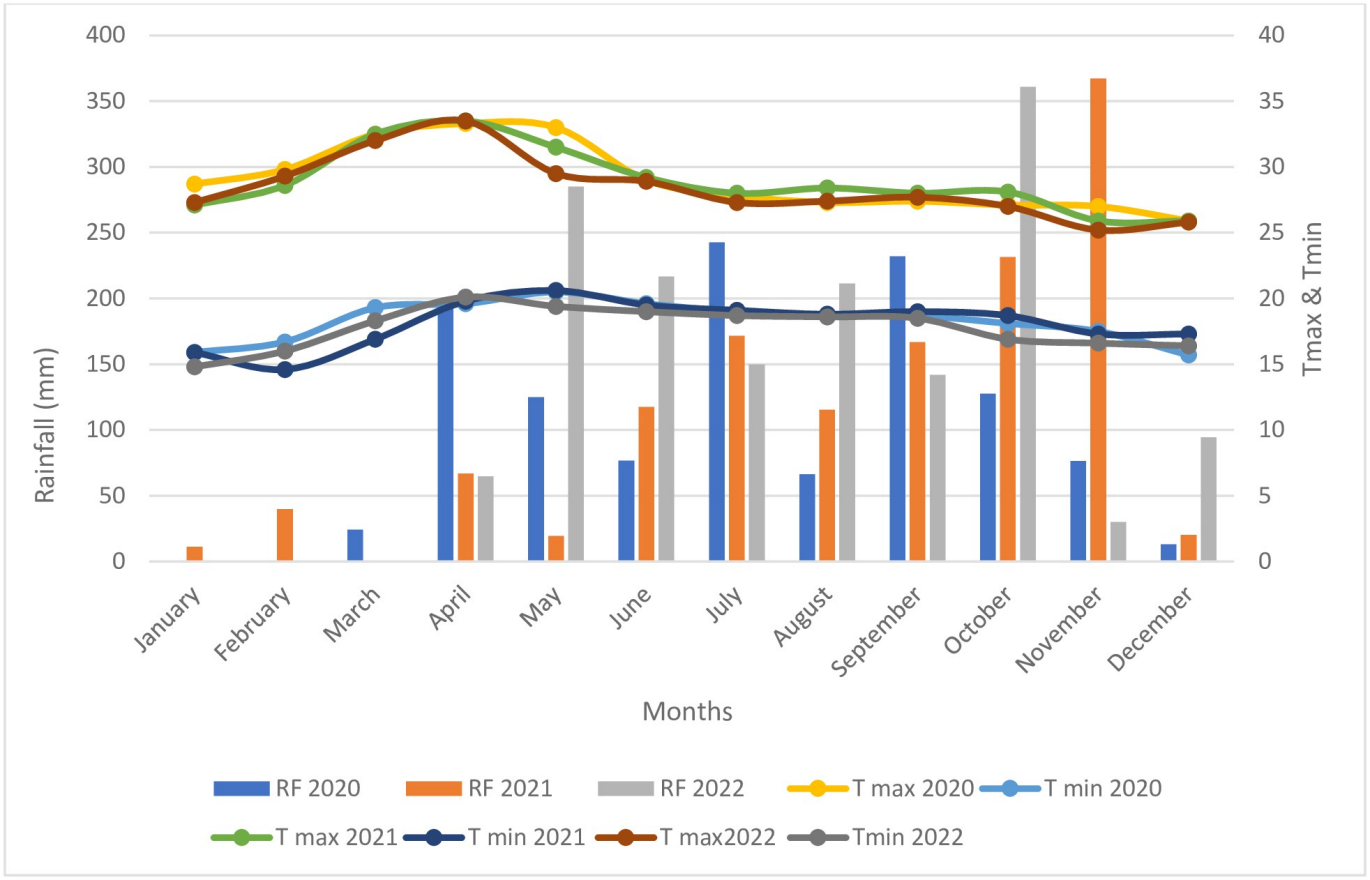
Variation in annual rainfall, maximum and minimum temperature during the field experiments from 2020–2022 (RF: Rainfall; T max: Temperature maximum; T min: Temperature minimum).

The research site exhibits a dry tropical savanna environment characterized by fairly hot summers and relatively cool winters. Three field studies were conducted at the F12 block of the Zonal Agricultural Research Station, University of Agricultural Sciences, Bangalore, Karnataka, India. The field site was permitted and approved by University of Agricultural Sciences, Bangalore. These studies included a fertility gradient experiment with fodder maize in 2020, a test crop experimentation with sweet corn in 2021 aimed at developing a fertilizer prescription equation, and a verification experimentation in 2022. The location of the research station is situated at 13° 04’ 55.2’’ N latitude, 77° 34’ 10.0’’ E longitude, with an altitude of 930 meters above mean sea level. The total amount of precipitation recorded in 2020 was 1180.0 mm which increased to. 1328.40 mm in 2021, and 1556.80 mm in 2022. In the years 2020, 2021 and 2022, the maximum temperature exhibited fluctuations within the ranges of 29.07 to 18.28°C, 28.89 to 18.13°C, and 28.41 to 17.78°C, respectively (Fig 1).

The well-drained red soil in the study location belonged to the taxonomically defined large group known as *Typic Kandic Paleustalfs* belonging to the fine mixed *Isohyperthermic* family. Soil samples were randomly collected from 0–15 cm depth from the experimental field before experimentation, and the composite was shade dried, processed and evaluated for chemical characteristics. The findings indicated that the pH value, electrical conductivity, organic carbon content, available nitrogen, available phosphorus and available potassium were measured at 6.13, 0.23 dS m^−1^, 5.10 g kg^-1^, 260.17 kg ha^−1^, 46.69 kg ha^−1^, and 134. kg ha^−1^, respectively.

### Fertility gradient experiment with fodder maize

A fertility gradient experiment was performed in 2020 to nullify the previous effects on soil fertility and create artificial fertility with respect to available NPK prior to testing crops [9]. Three identical strips were made and these strips were fertilized with three levels of N, P, and K (0-0-0, 150-135-200 and 300-270-400 kg ha^−1^, respectively). The fertilizer levels and layout of the fertility gradient experiment are presented in the S1 Fig. The standard dose of nitrogen fertilizers was fixed based on the recommended dose of fertilizers for fodder maize (150 kg ha^−1^). The phosphorus and potassium doses were fixed based on P and K fixing capacity (135 and 200 kg ha^−1^, respectively) of soil. An exhaust crop fodder maize (variety: African tall) was grown to stabilize the soil fertility and create an artificial fertility gradient. After the harvest of the fodder maize crop at 60 days after sowing, samples from the surface soil were collected from each strip. The soil samples were air-dried and ground to pass through a 2 mm sieve and the available N was estimated by alkaline KMnO_4_ method [10], available P by extraction with Bray’s extractant (*i*.*e*., 0.025 M HCl and 0.03 M NH_4_F) and determined calorimetrically by the ascorbic acid method [11] and available K by extraction with 1 N ammonium acetate (pH 7.0) and fed directly to flame photometer to assess the development of fertility gradient [12].

### Test crop experiment with sweet corn

The experimental study was carried out during the *rainy* season of 2021, with the primary objective of developing a target yield equations for sweet corn. The experiment involved splitting each fertility strip into three separate blocks based on manure., namely F_0_—without FYM (Farm Yard Manure), F_1_—the recommended dosage of FYM (10 t ha^-1^) and F_2_—double the recommended dose of FYM (20 t ha^-1^). Within these manure blocks, additional subdivisions were carried out, resulting in the creation of eight subplots. The subplots were specifically devised to allow a total of seven distinct combinations of NPK fertilizers, in addition to an absolute control. Overall, this experimental arrangement yielded a total of 24 treatments, consisting of 21 combinations of NPK and three control groups within each strip. Consequently, a total of 72 plots (24 × 3) were obtained across all three strips. The treatments included various amounts of nitrogen (0, 75, 150, and 225 kg ha^−1^), P_2_O_5_ (0, 37.50, 75 and 112.50 kg ha^−1^), and K_2_O (0, 18.75, 37.50, and 56.25 kg ha^−1^). In addition, three distinct FYM levels (0, 10 and 20 t ha^−1^) were included in the experiment. The layout and the amount of nutrients that were added are given in the S2 Fig.

Before sowing, initial soil samples were collected from 0–15 cm depth in each experimental plot, in accordance with the experimental design. These samples were analysed in the same manner as the gradient experiment to determine the levels of available nitrogen, phosphorus, and potassium. Before sowing, farm yard manure was applied 15 days beforehand. Three to four days prior to sowing, pre-soak irrigation was administered, and seeds were dibbled at a 45 cm x 30 cm spacing. As a base dose, half of the prescribed quantity of nitrogen, and the full amount of phosphorus and potassium were applied. The sources of nitrogen, phosphorus, and potassium were, in order, urea, single super phosphate and muriate of potash. The remainder of the nitrogen was administered as a top dressing in two equal portions, one at 30 days after sowing (DAS) and the other at 60 DAS. A steady supply of moisture was maintained around the plant’s root zone. All treatments included shallow cultivation at regular intervals to maintain the field free of weeds and to promote soil aeration and healthy root development, as well as pest and disease control measures. At maturity, the crop was harvested, and the produce of each plot was tallied. This yield information was expressed as tons per hectare (t ha^−1^) for analysis purposes.

#### Plant analysis

Plant samples were carefully collected from each plot and subjected to a systematic drying process. Initially, they were dried in the shade and subsequently in a hot air oven set at 65°C to ensure thorough desiccation. After this, the dried plant samples were finely ground using a Willey mill. The determination of nitrogen content in the plant samples was conducted through the micro Kjeldahl method [13]. For the preparation of a di-acid extract, a specific procedure outlined by Jackson (1973) [14] was followed for P and K analysis. This involved using a mixture of nitric acid (HNO_3_) and perchloric acid (HClO_4_) in a ratio of 9:4. Before the extraction, a pre-digestion step was carried out using 10 mL of HNO_3_ per gram of the plant sample. This di-acid extract was then employed for the assessment of Phosphorus (P) and Potassium (K) content in the plant samples. Phosphorus was quantified spectrophotometrically using the vanadomolybdate phosphoric acid yellow colour method [14]. Potassium, on the other hand, was estimated with flame photometer, following the procedure outlined by Jackson (1973) [14].

From the chemical analytical data, the uptake of each nutrient was calculated as shown below:

$$Nutrient\;upatke\;\left(\text{kg}\;{\text{ha}}^{-1}\right)=\frac{\left[Nutrient\;content\;\left(\%\right)\;X\;Dry\;weight\;\left(\text{kg}\;{\text{ha}}^{-1}\right)\right]}{100}$$

#### Basic parameters of targeted yield equations

The calculation of basic parameters such as nutritional requirement (NR), contribution of nutrients from the soil (CS), fertilizer (CF) and organic manure (COM) was performed [9], based on the data obtained from soil test values, crop dry matter yield and nutrient uptake.

$$NR\;(kg\;q^{-1})=\frac{Total\;Nutrient\;Uptake\;(kg\;{ha}^{-1})}{Cob\;yield\;(t\;{ha}^{-1})}$$

$$\%\;CS=\frac{Total\;nutrient\;uptake\;in\;control\;plot\;\left(kg\;{ha}^{-1}\right)}{Soil\;test\;value\;\left(Av.\;NPK\right)\;in\;control\;plot\;(kg\;{ha}^{-1})}\times 100$$

$$\%\;\text{CF}=\frac{\left[\text{Total}\;\text{nutrient}\;\text{uptake}\;\text{in}\;\text{treated}\;\text{plot}\;\left(\text{kg}\;{\text{ha}}^{-1}\right)-(\text{Soil}\;\text{test}\;\text{value}\;\text{in}\;\text{treated}\;\text{plot}\;\times \frac{\%\;\text{CS}}{100}\right]}{\text{Fertilizer}\;\text{nutrient}\;\text{dose}\;\text{applied}\;\text{in}\;\text{treated}\;\text{plot}\;\left(\text{kg}\;\text{h}{\text{a}}^{-1}\right)}\times 100$$

$$\%\;\text{C-OM}=\frac{\left[\text{Total}\;\text{uptake}\;\text{of}\;\text{NPK}\;\text{in}\;\text{organic}\;\text{plot}\;\left(\text{kg}\;{\text{ha}}^{-1}\right)—(\text{STV}\;\text{in}\;\text{organic}\;\text{plot}\;\times \frac{\%\;\text{CS}}{100}\right]}{\text{Quantity}\;\text{of}\;\text{organic}\;\text{manure}\;\text{added}\;\text{in}\;\text{organic}\;\text{plot}\;\left(\text{kg}\;\text{h}{\text{a}}^{-1}\right)}\times 100$$

By using the basic parameters, the fertilizer nutrient requisite for the targeted yield of sweet corn was calculated as follows [9]

**i**) **Fertilizer N**, **P and K alone**
$$FN=\left(\frac{NR}{CF}\times 100\times T\right)-\left(\frac{CS}{CF}\times SN\right)$$
$$FP=\left(\frac{NR}{CF}\times 100\times T\right)-\left(\frac{CS}{CF}\times SP\right)$$
$$FK=\left(\frac{NR}{CF}\times 100\times T\right)-\left(\frac{CS}{CF}\times SK\right)$$
**ii**) **Fertilizer N**, **P and K along with FYM**
$$FN=\left(\frac{NR}{CF}\times 100\times T\right)-\left(\frac{CS}{CF}\times SN\right)-\left(\frac{COM}{CF}\times OM\right)$$
$$FP=\left(\frac{NR}{CF}\times 100\times T\right)-\left(\frac{CS}{CF}\times SP\right)-\left(\frac{COM}{CF}\times OM\right)$$
$$FK=\left(\frac{NR}{CF}\times 100\times T\right)-\left(\frac{CS}{CF}\times SP\right)-\left(\frac{COM}{CF}\times OM\right)$$

Where, FN: Quantity of nitrogen to be added through fertilizers (kg ha^−1^); FP: Quantity of phosphorus to be added through fertilizers (kg ha^−1^); FK: Quantity of potassium to be added through fertilizers (kg ha^−1^) SN: Soil test value for available N (kg ha^−1^); SP: Soil test value for available P (kg ha^−1^) SK: Soil test value for available K (kg ha^−1^); COM: Contribution of nutrients from FYM; OM: Quantity of FYM applied (t ha^−1^).

### Verification trial with sweet corn

During the *rainy* season of 2022, a field experiment was undertaken to validate the STCR targeted yield equations that had been developed in the main experiment. The specific crop used in this experiment was sweet corn, with the variety Sugar 75. This process of validation considered important factors such as fresh cob and dry stover yield, percentage deviation from the predetermined target and economic factors. The experiment was designed with three replicates using a Randomized Block Design (RBD). The treatments comprised of, T_1_—STCR target 25 t ha^−1^ (NPK alone), T_2_—STCR target 25 t ha^−1^ (NPK + FYM), T_3_—STCR target 22 t ha^−1^ (NPK alone), T_4_—STCR 22 t ha^−1^ (NPK + FYM), T_5_—General Recommended dose (GRD), T_6_ –Soil fertility rating approach, T_7_—Absolute control.

An initial soil sample from the surface soil was collected prior to the sowing of sweet corn. Fertilizer prescription equations developed in test crop experiment on sweet corn were used to calculate the amount of fertilizer nutrients for achieving yield target of 25 and 20 t ha^−1^. The FYM used in this experiment had 0.58% total N, 0.15% total P and 0.54% total K. The crop was cultivated following established agronomic practices and allowed to reach full maturity. Upon maturity, the crop was harvested and the yield of fresh cob and dry stover was determined by assessing the net plot yield. The yield data was expressed in t ha^−1^. Additionally, samples of both cob and stover were collected from each treatment and subjected to analysis to determine their total NPK content. The procedure for this analysis was consistent with the methodology used in the test crop experiment. The uptake of NPK nutrients by the crop was calculated based on the results obtained from this analysis. The Response Yard Stick (RYS), percent deviation and Value Cost Ratio (VCR) were computed by using the standard formulae [9].


$$\text{RYS}=\frac{Yield\;response\;\left(\text{kg}\;{\text{ha}}^{-1}\right)}{Total\;nutrient\;applied\;\left(\text{kg}\;\text{h}{\text{a}}^{-1}\right)}$$

$$\text{Percent}\;\text{deviation}=\frac{\left[\text{Actual}\;\text{yield}\;\text{obtained}\;\left(\text{kg}\;{\text{ha}}^{-1}\right)-Targeted\;yield\;\left(\text{kg}\;{\text{ha}}^{-1}\right)\right]}{\;\text{Targeted}\;\text{yield}\;\left(\text{kg}\;\text{h}{\text{a}}^{-1}\right)}\;\times 100$$

$$\text{VCR}=\frac{\left[\text{Yield}\;\text{in}\;\text{treated}\;\text{plot}\;\left(\text{t}\;{\text{ha}}^{-1}\right)\;-Yield\;in\;control\;plot\;\left(\text{t}\;{\text{ha}}^{-1}\right)\right]}{\;\text{Cost}\;\text{of}\;\text{fertilizers}\;\text{and}\;\text{FYM}\;\text{applied}\;\text{to}\;\text{treated}\;\text{plot}}\times Cost\;t^{-1\;}of\;Cob$$

#### Nutrient use efficiency

Nutrient (N/P/K) use efficiency parameters *viz*., Recovery efficiency (RE), Partial factor productivity (PFP) and Reciprocal internal utilization efficacy (RIUE) were calculated using the following formulae [15]:

$$\text{RE}\left(kg\;{kg}^{-1}\right)=\frac{\left[\text{Nutrient}\;\text{uptake}\;\text{in}\;\text{treated}\;\text{plot}\;\left(\text{kg}\;{ha}^{-1}\right)-Nutrient\;uptake\;in\;control\;plot\;\left(\text{kg}\;{ha}^{-1}\right)\right]}{\text{Fertilizer}\;\text{nutrient}\;\text{applied}\;\left(\text{kg}\;{ha}^{-1}\right)}$$

$$PFP\;\left(kg\;{kg}^{-1}\right)=\frac{\left[Yield\;obtained\;in\;treated\;plot\;\left(kg\;{ha}^{-1}\right)\right]}{Fertilizer\;nutrient\;applied\;\left(kg\;{ha}^{-1}\right)}$$

$$\text{RIUE}\;(kg\;t^{-1})=\frac{\text{Nutrient}\;\text{uptake}\;\text{by}\;\text{cob}\;\left(\text{kg}\;{ha}^{-1}\right)}{\text{Cob}\;\text{yield}\;\left(\text{t}\;\text{h}{\text{a}}^{-1}\right)}$$

### Statistical analysis

The fertility gradient created was displayed in a box and whiskers plot and the main experiment data was summarized using descriptive statistics with IBM SPSS 16.0 software. Data recorded in verification experiments were analyzed using the ANOVA technique [16]. Treatment means were compared with a Tukey’s HSD test with a probability level of 0.05.

## Results

### Fertility gradient experiment

**Fig 2:**
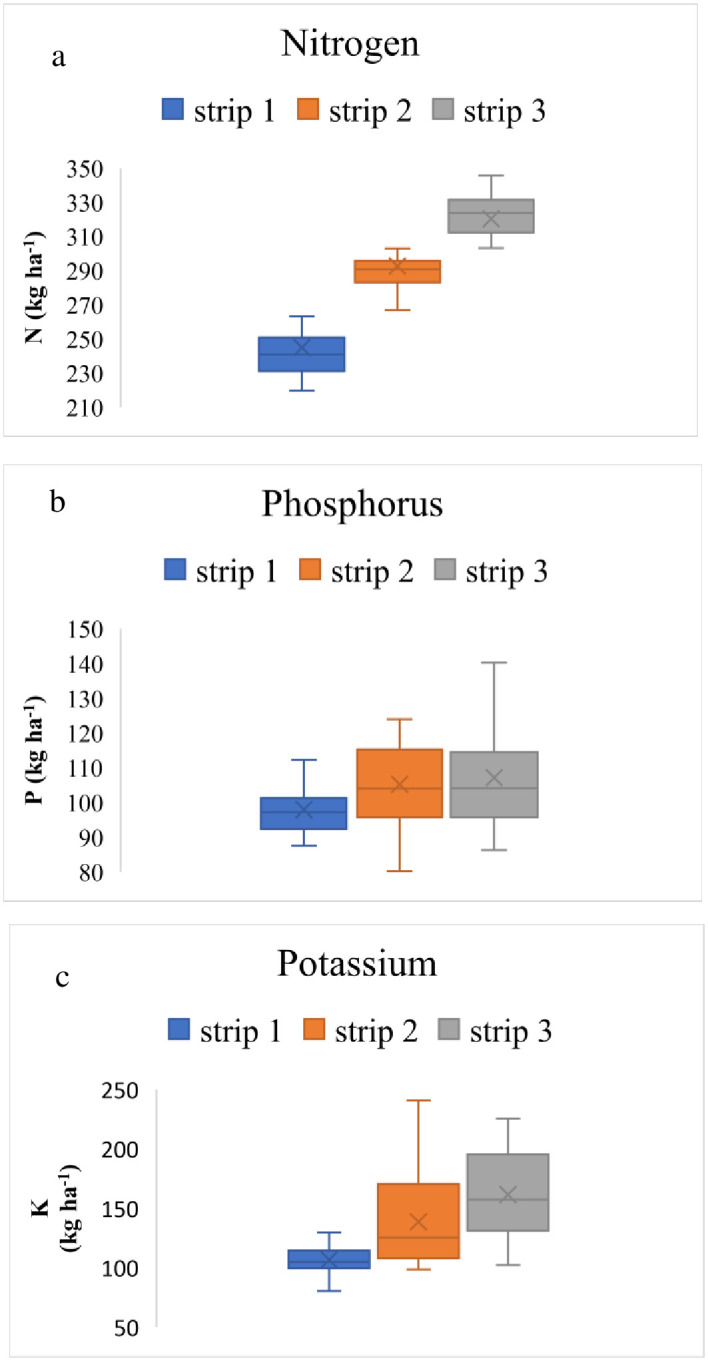
Box and whisker plots of the a) available nitrogen b) available phosphorus c) available potassium soil macronutrients (0–15 cm), after the soil fertility gradient experiment.

Gradient experiments were conducted to minimize the factors related to soil and other management practices that could affect crop yield. The fertility gradient was achieved by growing fodder maize, since maize is an exhaustive crop causing over-mining of plant nutrients, thus leaving relatively stable nutrient sinks in the soil that resulted in creating the fertility gradient. The variation in the mean soil test values for N, P_2_O_5_, and K_2_O in relation to the fertility strips is illustrated in Fig 2. The average concentrations of soil available N, P_2_O_5_, and K_2_O were determined in the strip I (244.97, 97.87, and 106.57 kg ha^-1^, respectively), strip II (292.56, 105.13, and 135.52 kg ha^-1^, respectively), and strip III (320.16, 107.09, and 161.46 kg ha^-1^, respectively) as shown in Fig 2. The nitrogen content of strip I is the lowest of the three. The interquartile range (IQR) signifies the central 50% of the data and is the narrowest, suggesting lower variability in the nitrogen concentration. Strip I has the most variability in nitrogen levels with an IQR of 32.2, while Strip III has the lowest with 19.04. The plot in strip I is symmetrical, resulting in an approximate skewness of zero. The skewness shows that some higher and lower values, respectively, are pushing the mean in the direction of strips II and III. In terms of the amount of P_2_O_5_ and K_2_O, the IQR is largest in strip III (64.66) for K_2_O and in strip II (31.57) for P_2_O_5_. The lowest IQR was seen in strip I for both P_2_O_5_ and K_2_O concentrations, with values of 24.69 and 30.00, respectively. The whisker and half-box are longer on the upper side of the median than on the lower side, resulting in a positively skewed distribution of K_2_O in all the strips. The distribution of P_2_O_5_ is positively skewed, while strip III is negatively skewed (Fig 2).

### Fresh cob yield and nutrient uptake of sweet corn under main experiment

**Fig 3:**
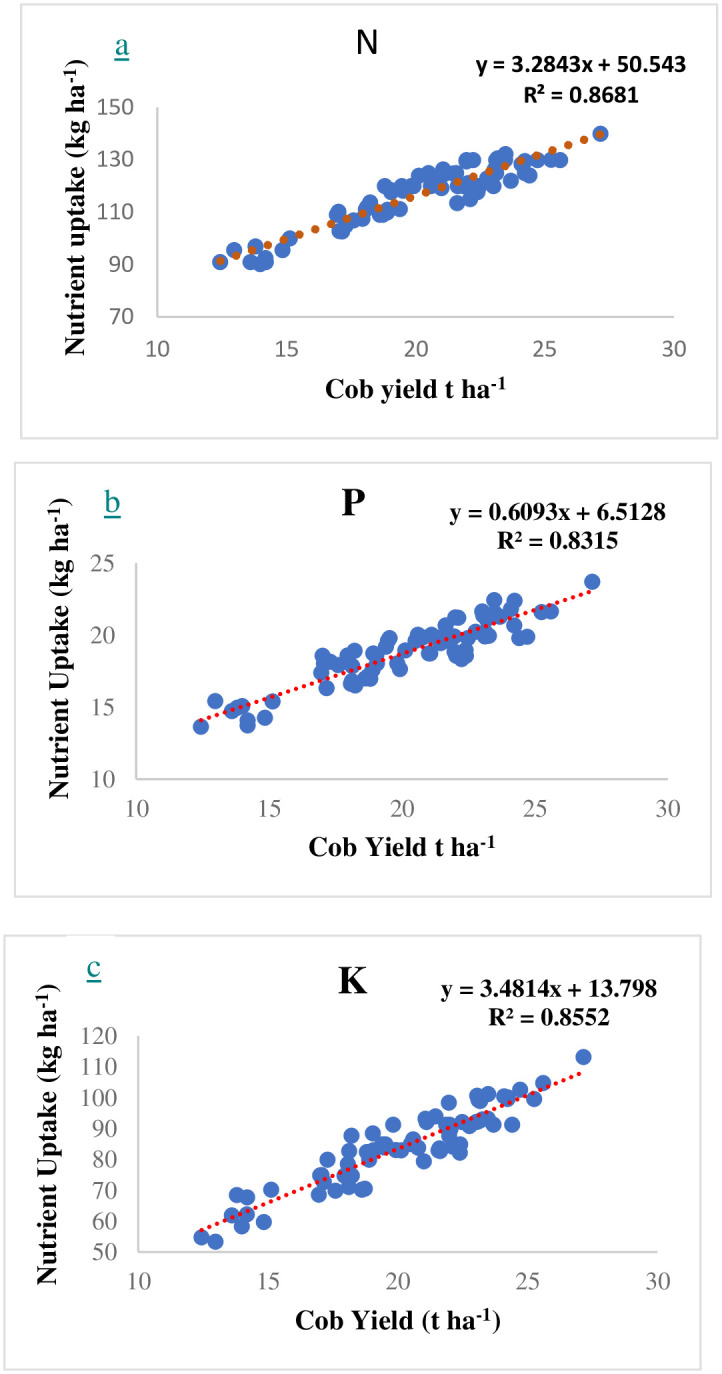
Linear correlations between cob yield and total uptake of a) N (a), a) P (b), and c) K (c) in sweet corn during the main experiment.

**Fig 4:**
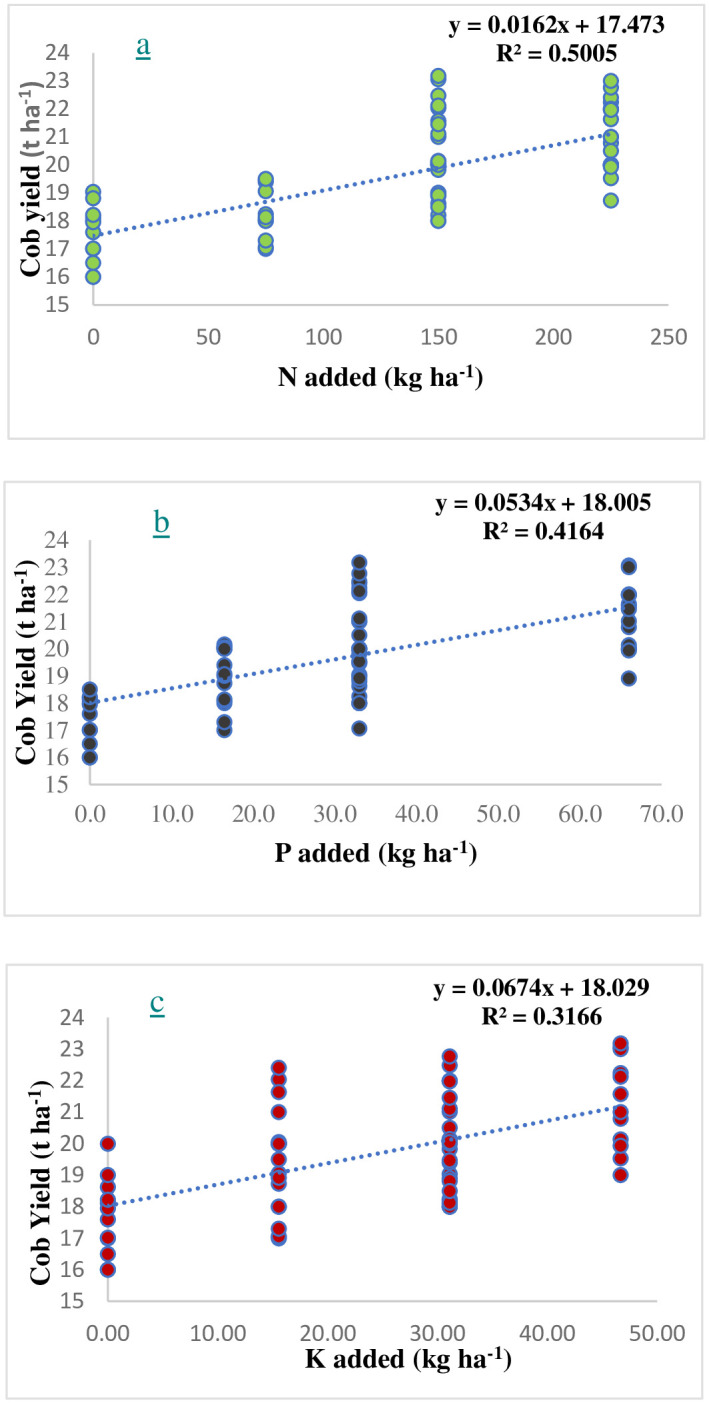
Linear correlations between a) N (a), b) P (b) and c) K (c) added and cob yield in sweet corn in the main experiment.

During *rainy* 2021, the STCR targeted yield equation for sweet corn was developed. The descriptive statistics for sweet corn fresh cob yield, dry stover yield and nutrient uptake are provided in Table 1. The mean fresh cob and dry stover yield followed the order strip III (20.88 and 10.98 t ha^-1^, respectively) > strip II (20.64 and 10.51 t ha^-1^, respectively) > strip I (18.98 and 10.04 t ha^-1^, respectively) with a variation of 20.18, 15.46 and 12.78 percent for fresh cob yield and 11.65, 10.51 and 12.07 percent for dry stover yield in strip I, II and III, respectively. Similar trend of results were noticed with respect to total uptake of N with the variation of 24.98, 26.44 and 15.53 percent; P with the variation of 27.29, 30.30 and 24.04 percent; K with variation of 18.86, 25.56 and 17.57 percent in strip I, II and III, respectively. A strong correlation (p < 0.001) was observed for the cob yield of sweet corn with total nitrogen uptake (r^2^ = 0.868), followed by total potassium uptake (r^2^ = 0.855) and total phosphorus uptake (r^2^ = 0.832) (Fig 3). Sweet corn cob yield exhibited a positive connection (p 0.001) with N dose (r^2^ = 0.500), followed by P and K doses (r^2^ = 0.416 and 0.316, respectively) (Fig 4). The data on yield, uptake and amount of fertilizer and FYM added for strip I, II, and III in provide in S1–S3 Tables, respectively.

**Table 1:** Descriptive statistics of yield and nutrient uptake under the main experiment of sweet corn.

Strips		Fresh cob yield (t ha^−1^)	Dry stover yield (t ha^−1^)	Total N uptake (kg ha^−1^)	Total P uptake (kg ha^−1^)	Total K uptake (kg ha^−1^)
I	Range	12.44–24.41	8.10–11.14	60.23–160.00	5.39–19.95	32.04–82.98
	Mean ± SD	18.98 ± 3.83	10.04±1.17	113.57±28.37	15.48±4.22	66.55±12.55
	(CV %)	20.18	11.65	24.98	27.29	18.86
II	Range	14.19–25.6	8.36–13.10	65.97–179.04	7.48–25.76	43.41–145.44
	Mean ± SD	20.64±3.19	10.51±1.29	122.31±32.34	17.96±5.44	90.16±23.135
	(CV %)	15.46	12.27	26.44	30.30	25.56
III	Range	14.85–27.16	7.98–13.91	95.63–190.16	11.11–37.41	48.64–126.43
	Mean ± SD	20.88±2.67	10.98±1.33	135.73±21.08	23.46±5.64	99.28±17.44
	(CV %)	12.78	12.07	15.53	24.04	17.57

Note: SD- standard deviation; CV (%)- co-efficient of variation.

#### Development of basic parameters

The targeted yield model encompasses the utilization of data pertaining to the yield of economic produce, the total uptake of NPK, initial soil test values, and the application rates of N, P, and K. The parameters employed in the formulation of fertilizer prescription equations for sweet corn consist of the nutrient requirements in kilograms per ton of economic produce (NR), the proportion of nutrients (NPK) obtained by the sweet corn crop from soil-available nutrients (%CS), the proportion of nutrients derived from fertilizer sources (%CF), and the percentage of nutrients obtained from organic manure sources (%COM). The primary data obtained from the test crop experiment, covering the use of both inorganic fertilizers alone and the integrated approach, were separately computed and presented in Table 2.

**Table 2:** Basic parameters for calculating fertilizer requirement, with and without FYM, for the targeted yield of sweet corn.

Parameters	NPK alone			NPK + FYM		
	N	P	K	N	P	K
**NR (kg t** ^**−1**^ **)**	5.85	0.87	4.31	6.07	0.92	4.33
**CS%**	29.53	7.73	44.03	29.53	7.73	44.03
**CF%**	39.72	23.58	104.28	42.25	24.29	94.48
**COM%**	-	-	-	0.871	0.35	0.64

Note: NR: Nutrient requirement; CS: Contribution of nutrient from soil; CF: Contribution of nutrients from fertilizers; COM: Contribution of nutrients from organic manure

The nutrients required to produce one ton of fresh cob yield (NR) were 5.85 kg, 0.87 kg and 4.31 kg of N, P and K, respectively under the STCR NPK alone approach and 6.07 kg, 0.92 kg and 4.33 kg for N, P and K, respectively under STCR NPK+FYM approach. The contribution of nutrients from the soil is expressed as the capacity of the crop to extract nutrients from the soil. The percent contribution of N, P and K from soil was 29.53, 7.73 and 44.03%, respectively under both NPK alone and STCR NPK+FYM approaches. The nutrient contribution from fertilizers under NPK alone and STCR NPK+FYM approach were 39.72 and 42.25%for N, 23.58 and 24.29%for P, 104.28 and 94.48% for K, respectively towards the uptake by sweet corn.

#### Formulating prescription equation for sweet corn production using STCR NPK alone and STCR NPK+FYM approach

By using the data from the above basic parameters, the fertilizer prescription equations were developed separately for purely STCR NPK alone and STCR NPK+FYM approaches and are presented below.

**STCR- NPK alone equations**.
F.N.=14.74T−0.744SN (1)
F.P.=1.61T−0.328SP (2)
F.K.=3.45T−0.422SK (3)
**STCR- NPK+FYM equations**.
F.N.=14.363T−0.699SN−0.871(OM) (4)
F.P.=1.644T−0.319SP−0.349(OM) (5)
F.K.=3.824T−0.388SK−0.637(OM) (6)

Where, FN, FP and FK are fertilizer N, P and K in kg ha^−1^ respectively; T is the yield target in t ha^−1^; SN, SP and SK are available soil nutrients as KMnO_4_-N, Bray’s-P and NH_4_OAc-K in kg ha^−1^ respectively and OM is the amount of farm yard manure (organic manure) added in t ha^−1^.

### Fresh cob and dry stover yield under verification trial

The fresh cob and dry stover yield of sweet corn varied from 6.86 t ha^−1^ and 10.29 t ha^−1^, respectively in absolute control to 23.21 t ha^−1^ and 34.81 t ha^−1^, respectively in STCR NPK alone approach for the targeted yield of 25 t ha^−1^ (Table 3). Among the treatments, STCR NPK+FYM approach for the targeted yield of 25 t ha^−1^ recorded significantly higher cob yield (23.38 t ha^−1^) and dry stover yield (35.07 t ha^−1^) which is on par with STCR NPK alone approach for the yield target of 25 t ha^−1^, STCR NPK+FYM approach for the targeted yield of 22 t ha^−1^ and SFR approach of fertilizer recommendation. The cob and dry stover yield of GRD were significantly lower compared to STCR NPK+FYM approach for the targeted yield of 25 t ha^−1^ proving the superiority of STCR approach. The STCR NPK + FYM approach for the targeted yields 25 t ha^-1^ enhanced the cob yield to an extent of 8.64% over GRD and 3.04% over SFR approach.

**Table 3:** Influence of different approaches of nutrient recommendations on yield, % deviation, response yard stick (RYS) and value cost ratio (VCR) of sweet corn.

Treatment	Nutrients added (kg ha^−1^)			Fresh cob yield (t ha^−1^)	Dry stover yield (t ha^−1^)	% deviation	RYS	VCR	Nutrient uptake (kg ha^−1^)		
	N	P	K						N	P	K
T_1_	210.21	15.70	16.69	23.21^ab^	34.81^ab^	-7.16	61.47	75.27	181.04^ab^	13.79^ab^	119.44 ^ab^
T_2_	184.02	13.49	10.43	23.38^a^	35.07^a^	-6.49	72.69	9.28	182.35^a^	14.71^a^	120.31 ^a^
T_3_	146.36	9.67	11.92	21.46^b^	32.19^b^	-2.45	79.92	100.89	167.4^b^	12.75^b^	110.44^b^
T_4_	136.95	9.13	6.46	22.34^ab^	33.51^ab^	1.55	93.55	8.97	174.26^ab^	13.93 ^ab^	114.96 ^ab^
T_5_	150.00	33.00	33.20	21.52^b^	32.28^b^	-2.18	55.32	7.61	167.86^b^	12.47^b^	110.75^b^
T_6_	187.50	27.50	33.20	22.69^ab^	34.03^ab^	3.13	54.58	8.28	176.96^ab^	13.48 ^ab^	116.75 ^ab^
T_7_	-	-	-	6.86^c^	10.29^c^	-68.82	-	-	68.59^c^	5.49^c^	35.29^c^
Significance level				*	*				*	*	*

Note: RYS: Response Yard Stick; VCR: Value Cost Ratio; T_1_- STCR 25 t ha^−1^ (NPK Alone); T_2_- STCR 25 t ha^−1^ (NPK+FYM); T_3_- STCR 22 t ha^−1^ (NPK Alone); T_4_- STCR 22 t ha^−1^ (NPK+FYM); T_5_- General recommended dose; T_6_- Soil fertility rating; T_7_- Absolute control.

*Significance at 5% level: Values followed by the same letter are not different at a 5% probability level

#### Percent deviation and response yard stick

The percent deviation in this study indicates how the crop yield varies from the predetermined or genetically expected potential (Table 3). In the case of the STCR approach targeting 25 t ha^−1^ and 22 t ha^−1^ through an STCR NPK alone approach, the percent deviation was negative (-7.16% and -2.45%, respectively), signifying that the actual yield fell below the fixed target. In contrast, the STCR NPK+FYM approach aiming for a 25 t ha^−1^ yield had a negative deviation of -6.49%, indicating it was close to the target. However, the STCR NPK+FYM approach targeting 22 t ha^−1^ and the soil fertility rating approach both showed positive deviations (1.55% and 3.13%, respectively), suggesting that they exceeded the target yield expectations. Both the soil fertility rating and general recommended dose of fertilizers recommendation approaches had lower RYS values, namely 54.58 kg kg^−1^ and 55.32 kg kg^−1^, respectively, which were shown to be inferior to the STCR treatments.

#### Value cost ratio

The cost-benefit analysis, as measured by the Value Cost Ratio (VCR), revealed notable differences in various fertilizer application approaches. Specifically, when fertilizer nutrients were applied using the STCR NPK alone approach to achieve target yields of 25 t ha^−1^ and 22 t ha^−1^, high VCR values of 75.27 and 100.89, respectively were achieved, indicating favorable returns on investment. In contrast, the application of nutrients based on the GRD and the SFR approach resulted in lower VCR values, specifically 7.61 and 8.28, respectively, when compared to the STCR approaches for fertilizer recommendation. However, it’s worth noting that the STCR NPK+FYM treatments recorded lower VCR values than the STCR NPK alone treatments, suggesting that the STCR NPK+FYM approach might have some economic trade-offs despite its higher yield potential.

#### Nutrient uptake

The uptake of NPK by sweet corn differed significantly due to the application of fertilizers based on different approaches of fertilizer recommendation (Table 3). The higher nitrogen, phosphorus and potassium uptake of 182.35, 14.71 and 120.31 kg ha^−1^ respectively, was recorded in treatment with the application of fertilizer nutrients based on STCR approach for the targeted yield of 25 t ha^−1^ through NPK+FYM approach which was on par with STCR NPK alone approach with the targeted yield of 25 t ha^−1^ (181.04, 13.79 and 119.44 kg ha^−1^ respectively), STCR NPK+FYM approach with the targeted yield of 22 t ha^−1^ (174.26, 13.93 and 114.96 kg ha^−1^ respectively) and SFR of fertilizers recommendation (176.96, 13.48 and 116.75 kg ha^−1^ respectively). The significant difference was noticed with STCR NPK alone approach with a targeted yield of 22 t ha^−1^ (167.4, 12.75 and 110.44 kg ha^−1^ respectively) and GRD. Whereas, lower uptake of 68.59, 5.49 and 35.29 kg ha^−1^ respectively of nitrogen, phosphorus and potassium was noticed in absolute control plots.

#### Nutrient use efficiency

Nutrient Use Efficiency (NUE) in the production of sweet corn is shown in Table 4. as a result of various nutrient management approaches. These nutrient management practices led to variations in NUE for nitrogen (REN), phosphorus (REP) and potassium (REK), with improvements observed in the STCR treatments compared to the SFR approach and the GRD. The highest REN, REP and REK values were achieved with the STCR approach, specifically when targeting a lower yield of 22 t ha^−1^ through an NPK+FYM mode (0.772 kg kg^−1^, 0.925 kg kg^−1^ and 13.342 kg kg^−1^, respectively). In contrast, the GRD and SFR resulted in lower REP and REK values (0.211 and 2.273 kg kg^−1^ for the GRD, and 0.291 and 2.454 kg kg^−1^ for the SFR.

**Table 4:** Nutrient use efficiency in sweet corn as influenced by different approaches of fertilizer recommendation.

Treatment	Nitrogen use efficiency			Phosphorus use efficiency			Potassium use efficiency		
	RE	PFP	RIUE	RE	PFP	RIUE	RE	PFP	RIUE
	(kg kg^−1^)		kg t^−1^	(kg kg^−1^)		kg t^−1^	(kg kg^−1^)		kg t^−1^
**T**_**1**_	0.535	2.76	1.986	0.529	16.26	0.337	5.041	28.85	1.628
**T**_**2**_	0.618	3.18	2.021	0.684	19.06	0.357	8.149	46.50	1.754
**T**_**3**_	0.675	3.67	1.962	0.751	24.42	0.337	6.303	37.35	1.635
**T**_**4**_	0.772	4.08	1.985	0.925	26.92	0.354	12.342	71.81	1.631
**T**_**5**_	0.662	3.59	1.917	0.211	7.17	0.329	2.273	13.45	1.511
**T**_**6**_	0.578	3.03	1.950	0.291	9.08	0.337	2.454	14.18	1.543
**T**_**7**_			2.500			0.454			1.426

Note: T_1_- STCR 25 t ha^−1^ (NPK Alone); T_2_- STCR 25 t ha^−1^ (NPK+FYM); T_3_- STCR 22 t ha^−1^ (NPK Alone); T_4_- STCR 22 t ha^−1^ (NPK+FYM); T_5_- General recommended dose; T_6_- Soil fertility rating; T_7_- Absolute control. RE: Recovery Efficiency; PFP: Partial Factor Productivity; RIUE: Reciprocal Internal Utilization Efficiency

A similar trend was observed in Partial Factor Productivity (PFP), with the highest PFP for nitrogen (PFPN), phosphorus (PFPP) and potassium (PFPK) reported in the STCR treatment targeting 22 t ha^−1^ through an NPK+FYM approach (4.08 kg kg^−1^, 26.92 kg kg^−1^ and 71.81 kg kg^−1^, respectively). These values were comparable to the other STCR treatments, the general recommended dose (3.59, 7.17 and 13.45 kg kg^−1^, respectively) and the soil fertility rating approach (3.03, 9.08 and 14.18 kg kg^−1^, respectively). In summary, the trend in Reciprocal Internal Use Efficiency (RIUE) followed the order RIUEK > RIUEP > RIUEN.

## Discussion

### Fertility gradient experiment

The primary objective of conducting the fertility gradient experiment was to establish a substantial range of variability in terms of soil-available N, P and K content. This variation was intentionally generated before initiating test crop trials involving sweet corn. The varying application rates of fertilizers, manures and the inherent soil fertility were the key factors contributing to the observed yield discrepancies in different segments of fodder maize.. Maize is an exhaustive crop causing over mining of plant nutrients, thus leaving relatively stable nutrient sinks in the soil that resulted in creating the fertility gradient [17, 18]. However, certain nutrients remain relatively stable in the soil due to their role as enduring nutrient reservoirs. This phenomenon led to the creation of the fertility gradient within the experimental setup. Elevated Soil Test Values (STVs) of nitrogen (N) observed in strip III could be attributed to the application of twice the amount of NPK fertilizers in comparison to the control, along with the utilization of single-fold doses [19]. As a result of graded fertilization, strip III showed the highest STVs of P and K [19]. In addition, P’s fixation in soil due to its immobile nature, might be the reason of its higher STVs [20]. The variability of N (7.07–10.20%) was considerably lower than P (7.59–16.73%) and K (10.00–16.58%) content mainly because N was lost through denitrification, leaching, and ammonia volatilization (particularly in rice crop) [21].

### Main experiment

The variations established within the gradient experiment, coupled with the application of varying quantities of nitrogen, phosphorus and potassium fertilizers, exerted a significant influence on the yield and nutrient uptake of sweet corn during the main crop experiment. Notably, the strip with higher fertility (strip III) showed the highest yield of sweet corn cobs. This outcome was attributed to the augmented nutrient application resulting from both the varied fertilizer quantities and the outcomes of the gradient experiment, which contributed to an elevated fertility status [22]. Across all strips (I/II/III), plots that received a combination of NPK fertilizer and farm yard manure (FYM) exhibited superior yields compared to plots treated solely with NPK fertilizer. This enhanced yield could be attributed to the direct impact of nutrient decomposition and mineralization from FYM, as well as the indirect effects stemming from the rhizosphere, which bolstered microbial activity and subsequently influenced nutrient availability [23, 24]. Similar to that, strip III showed the higher total nutrient uptake, followed by strips II and I. This might be attributed to higher yield caused by the application of a higher dose of fertilizer for the high fertility strips, making these nutrients more readily available for crop uptake [25]. Achieving a suitable nitrogen (N) application level, along with increased absorption and accumulation, contributed to enhanced production and uptake of nitrogen, phosphorus and potassium (NPK) [26]. It is plausible that the III strip contained an adequate supply of N fertilizer, potentially facilitating a favorable nitrogen uptake. The possible reason behind the highest uptake of phosphorus in strip III was attributed to better root proliferation, having a graded P application [27, 28]. The higher dose of N application stimulated the vegetative and root foraging capacity, meaning the crops require additional P and K, and increased the phosphorus uptake in the crops [29]. A similar trend was noticed with respect to potassium and might be attributed to the higher application of fertilizer potassium [30].

Soil test calibration was conducted to obtain specific yield objectives for sweet corn using a targeted yield model. This approach aimed to optimize fertilizer quantities through the integration of pre-sowing soil test values, the overall nutrient uptake and the doses of fertilizer N, P, K and organic manure (in the form of farm yard manure—FYM) that were applied. The fundamental data essential for formulating the targeted yield equations included calculations for nutrient requirements (NR), the contribution of nutrients derived from the soil (CS), those from fertilizer (CF) and those from organic manure (COM). These computations were performed for nitrogen, phosphorus and potassium (NPK) based on data gathered from the main experiment. The NR for N was 2.94 and 1.13 times higher than P and K, respectively under inorganics and 2.89 and 1.17 times higher than P and K, respectively under integrated approach. Nutrient requirement follows the trend of N > K > P, as it is mainly due to the interaction of the genotype of the variety with its surrounding environment in terms of nutrient availability, soil moisture and soil temperature [23, 31]. The contribution of nutrients from soil follows the trend K > N > P which might be due to the preferential absorption nature of crops [31]. The order of percentage contribution of fertilizer nutrients towards uptake by the crop thereby to yield under inorganic and integrated approach was K > P> N. The proportion of nutrient contribution originating from fertilizers surpassed that of the soil, mainly due to the faster and elevated availability of nutrients in an inorganic form from the fertilizers. Notably, the integrated approach resulted in a greater nutrient contribution from fertilizers compared to the single use of inorganic fertilizers. Intriguingly, an interesting observation emerged wherein the contribution of potassium (K) from fertilizers exceeded 100%. This anomaly was attributed to the interplay of higher doses of nitrogen (N) and phosphorus (P) at a specific K dose, alongside the primary effect of the initial K application in treated plots. This interaction prompted the release of soil-bound K and consequently led to increased crop uptake from the native soil sources in the presence of the applied K [32]. Contribution from organic carbon was calculated with the help of data using the FYM treated and control plots, and it followed the order N>P > K [33]. The nutrient supply through a native source in all the plots and their interaction effects might be the reason that the addition of CS, COM, CF under inorganics and CF under integrated approach was not equal in percentage [34].

### Verification trial

Fertilizer response pertains to the functional correlation between the increase in crop yield and the addition of fertilizer nutrients. This relationship can be graphically depicted or represented mathematically through an equation. Consequently, the formulation of fertilizer recommendations using a yield-targeting strategy holds a distinct characteristic. This approach not only prescribes fertilizer quantities based on soil test results but also outlines the potential yield level attainable by farmers if effective agronomic practices are employed in cultivating the crop [31].

The efficacy of the formulated targeted yield equations was assessed by comparing them with alternative methods of fertilizer recommendation. Notably, the treatment utilizing the STCR target of 25 t ha^−1^ through an STCR NPK+FYM approach demonstrated superior fresh cob and dry stover yields. This outcome can be attributed to the well-balanced nutrient application strategy based on soil analysis and encompassing considerations such as nutrient depletion caused by crops, initial soil fertility levels, nutrient efficacy present in the soil and those added through fertilizers [17] and effectiveness of targeted yield methodologies adapted in fulfilling the crop’s nutrient requirements [35]. These factors might have provided the optimum nutrients at the optimum time for better uptake and ultimately resulted in higher dry matter and yield [36].

The enhanced yield achieved through the STCR NPK+FYM approach at a higher target, in contrast to the STCR NPK alone approach, can primarily be attributed to the utilization of elevated fertilizer quantities. These increased doses likely facilitated the rapid release of nutrients in forms easily absorbed by the plants, consequently fostering improved growth and yield of sweet corn. The lower yield in general recommended dose could be attributed to the imbalanced fertilization without considering the crop need and contribution of nutrients from soil, fertilizer and FYM. The percent deviation of yield was within ± 10 percent variation proving the validity of the developed equations [17]. The yield targeting with NPK+FYM approach (STCR approach with target of 22 t ha^−1^) recorded relatively higher percent achievement than that aimed under their respective NPK alone treatment because of the higher yields achieved in the STCR NPK+FYM approach. It is also evident from the data that lower yield targets were better achieved than the higher one [37, 38]. Among the targets tried, targeting for 22 t ha^−1^ with STCR NPK+FYM approach recorded relatively higher RYS (93.55) than their respective inorganic approach with target of 22 t ha^−1^ (79.92) though it has recorded higher yields. This might be due to the better use efficiency of applied nutrients at low yield target levels [22]. Likewise, STCR NPK+FYM treatments recorded higher RYS when compared to their respective NPK alone treatments. The relatively higher RYS recorded under STCR treatments when compared to GRD (55.32) and soil fertility rating approach (54.58), might be due to balanced supply of nutrients from fertilizers, efficient utilization of applied fertilizer nutrients in the presence of organic sources and the synergistic effect of the conjoint addition of various sources of nutrients [8]. A similar trend of the superiority of STCR-integrated approach over farmer’s practice and the blanket recommendation for maize-tomato sequence [39].

The higher value cost ratio (VCR) under STCR NPK alone approach for the targeted yield of 25 t ha^−1^ and 22 t ha^−1^ could be mainly due to the application of required dose of NPK fertilizer without FYM associated with higher yields [35]. Even though higher yields were recorded in STCR NPK+FYM approach, the VCR was very low mainly due to high cost of FYM [34].

The higher uptake of nitrogen, phosphorus and potassium in STCR approach for the targeted yield of 25 t ha^−1^ through NPK+FYM could be attributed to the higher yield associated with a higher dose of NPK fertilizers compared to other treatments which might have helped in better availability of these nutrients in the vicinity of plant roots that might have increased the uptake [31]. Similarly, the higher yield with STCR approach compared to the general recommended dose could be attributed to the balanced application of nutrients considering the crop requirement and contribution from soil, fertilizer and FYM based STCR treatments [22]. It might be also due to the fact that the balanced use of various plant nutrient sources results in proper absorption, translocation and assimilation of nutrients, ultimately increasing the dry matter accumulation and nutrient contents of crop [40].

The higher use efficiency of nutrients in STCR approach with lower targets could be attributed to the higher yield with lower rates of fertilizer application as per the requirement of the crop [18]. The RIUE for nitrogen, phosphorus and potassium was enhanced markedly with STCR based nutrient management practices, compared to other approaches of fertilizer recommendation might be due to the balanced supply and efficient utilization of nutrients due to the synergistic effects of fertilizers and the applied FYM.

## Conclusion

The fertilizer prescription equations developed through STCR approach is a promising option to enhance the yield of sweet corn under varied soil fertility levels of *Alfisols* of Southern India. Results of the experiment demonstrated a marked response of sweet corn to the application of NPK fertilizers. The nutrient required to produce one ton of sweet corn cob yield were 5.85 kg, 0.87 kg and 4.31kg for N, P and K, respectively under the STCR NPK alone approach and 6.07 kg, 0.92 kg and 4.33 kg for N, P and K, respectively under STCR NPK+FYM approach. The magnitude of response was better when NPK fertilizers were integrated with FYM application based on STCR approach for the yield target of 25 ha^-1^and 30 t ha^-1^ relative to sole application of NPK fertilizers, general recommended dose and soil fertility rating approach. The percent achievement of the targeted yield was within ±10% variance at both targets, demonstrating the validity of the equations for prescribing fertilizer dose for sweet corn. It is important to acknowledge that the Value Cost Ratio exhibited a greater value in the STCR NPK+FYM method, principally attributed to the increased expenditure associated with farm yard manure. Hence, it is recommended to advocate for the use of STCR-based treatments, as they prioritize the significance of locally generating compost or farm yard manure. Promoting the practice of farmers producing their own compost or farm yard manure can yield several benefits, including cost reduction in production, sustained soil fertility and ultimately, improved economic returns for farmers.

## Supporting information

S1 Fig Layout and details of fertility gradient experiment of fodder maize crop. Were, **1:** 50% of recommended dose fertilizer. **2:** 100% of recommended dose fertilizer. **3:** 150% of recommended dose fertilizer.
(TIF)

S2 Fig The layout and the nutrients level tried under main experiment. (TIF)

S1 Table Details of treatment structure, yield, uptake and initial soil analytical data for sweet corn during in strip-I. (PDF)

S2 Table Details of treatment structure, yield, uptake and initial soil analytical data for sweet corn during in strip-II. (PDF)

S3 Table Details of treatment structure, yield, uptake and initial soil analytical data for sweet corn during in strip-III. (PDF)

## Abbreviations

AE: Agronomic nutrient use efficiency
CF: Contribution from Fertilizer
COM: Contribution from organic Manure
CS: Contribution from soil
GRD: General Recommended Dose
K: Potassium
N: Nitrogen
NR: Nutrient Requirement
P: Phosphorus
RE: Recovery Efficiency
RIUE: Reciprocal Internal Utilization Efficiency
RYS: Response Yard Stick
SFR: Soil Fertility Rating
STCR: Soil Test Crop Response
VCR: Value cost ratio
