# Supplementary figures and images for: Inductive cum targeted yield model-based integrated fertilizer prescription for sweet corn (Zea mays L. Saccharata) on Alfisols of Southern India
Source: PLoS One. 2024 Aug 26;19(8):e0307168. doi: 10.1371/journal.pone.0307168 (PMC11346652; doi:10.1371/journal.pone.0307168)

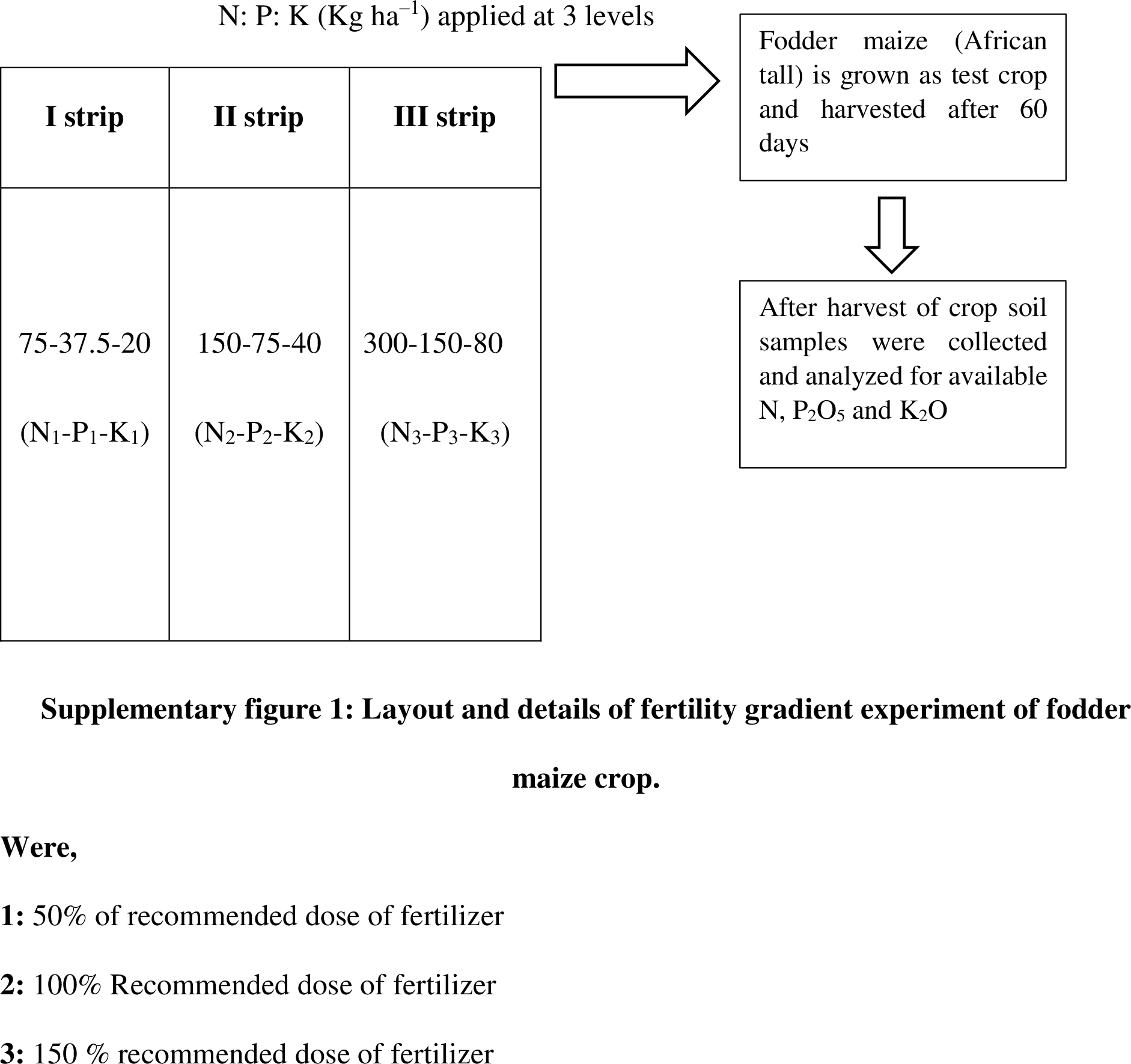

Supplement: S1 Fig — Were, 1: 50% of recommended dose fertilizer. 2: 100% of recommended dose fertilizer. 3: 150% of recommended dose fertilizer. (TIF) [file pone.0307168.s001.tif]

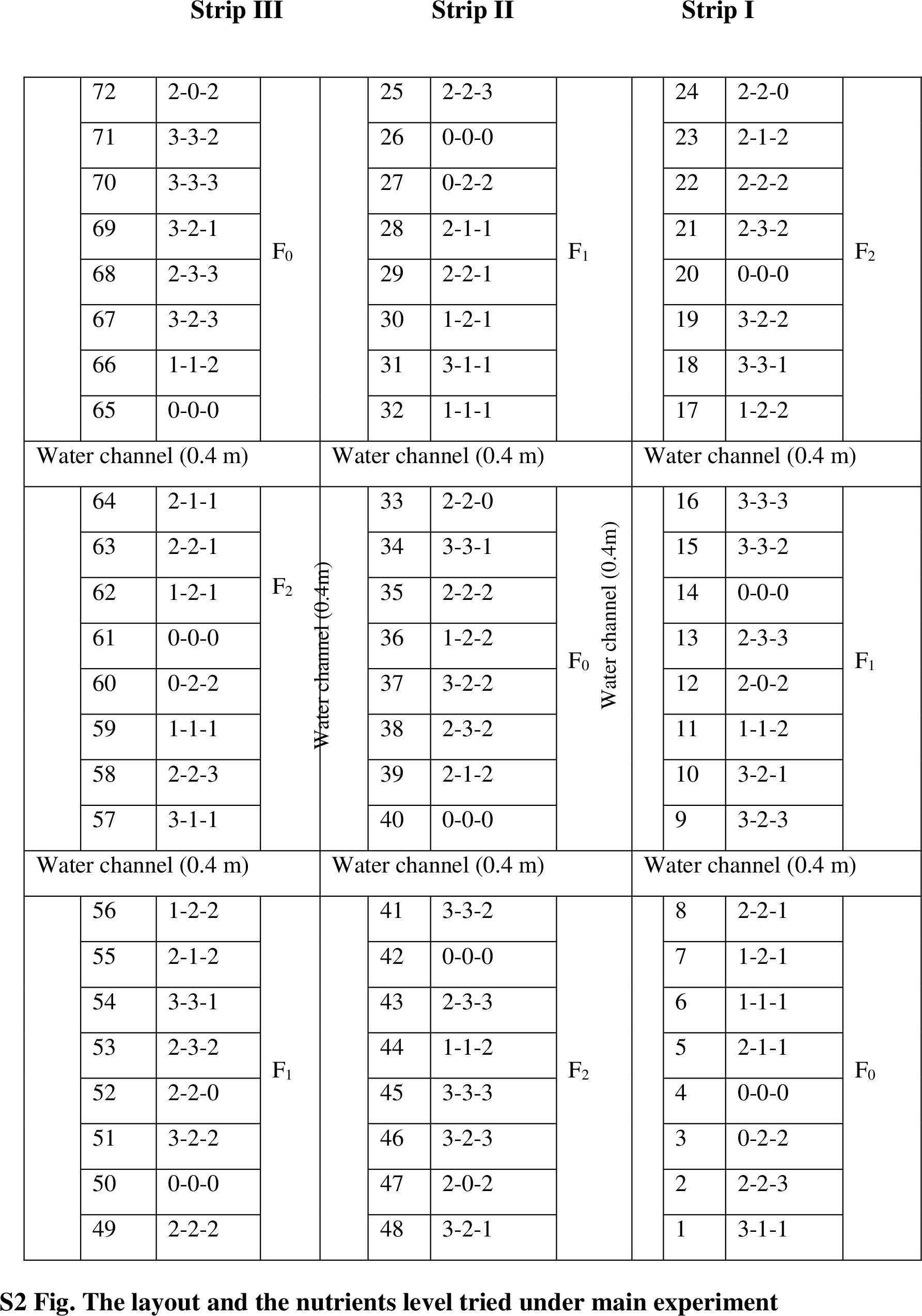

Supplement: S2 Fig — (TIF) [file pone.0307168.s002.tif]
