# Supplementary material for: Inductive cum targeted yield model-based integrated fertilizer prescription for sweet corn (Zea mays L. Saccharata) on Alfisols of Southern India
Source: PLoS One. 2024 Aug 26;19(8):e0307168. doi: 10.1371/journal.pone.0307168 (PMC11346652; doi:10.1371/journal.pone.0307168)
Supplement: S1 Table — (PDF) [file pone.0307168.s003.pdf]

## S1 Table: Details of treatment structure, yield, uptake and initial soil analytical data for sweet corn during in strip-I

| Sl No | Treatment | Yield ($t\ ha^{-1}$) | | Initial soil available N-P-K status ($kg\ ha^{-1}$) | | | Total uptake of N-P-K by crop ($kg\ ha^{-1}$) | | | Fertilizer N-P-K applied ($kg\ ha^{-1}$) | | | FYM applied ($t\ ha^{-1}$) |
|---|---|---|---|---|---|---|---|---|---|---|---|---|---|
| | | Cob yield | Straw yield | N | $P_2O_5$ | $K_2O$ | N | $P_2O_5$ | $K_2O$ | N | $P_2O_5$ | $K_2O$ | |
| $F_0$ 1 | 3 1 1 | 18.73 | 8.38 | 295.68 | 96.32 | 90.25 | 96.07 | 24.09 | 61.03 | 225 | 37.5 | 18.75 | 0.00 |
| 2 | 2 2 3 | 16.96 | 8.29 | 246.4 | 101.23 | 94.25 | 83.65 | 29.50 | 72.65 | 150.00 | 50.00 | 56.25 | 0.00 |
| 3 | 0 2 2 | 12.98 | 10.25 | 234.08 | 101.23 | 100.6 | 95.63 | 45.07 | 64.25 | 0.00 | 75.00 | 37.50 | 0.00 |
| 4 | 0 0 0 | 12.44 | 9.58 | 250.36 | 98.55 | 80.26 | 60.23 | 12.25 | 38.60 | 0.00 | 0.00 | 0.00 | 0.00 |
| 5 | 2 1 1 | 14.20 | 8.31 | 246.4 | 88.63 | 101.23 | 78.04 | 24.56 | 81.56 | 150.00 | 37.50 | 18.75 | 0.00 |
| 6 | 1 1 1 | 13.61 | 9.53 | 240.8 | 97.56 | 113.36 | 83.95 | 28.50 | 74.56 | 75.00 | 37.50 | 18.75 | 0.00 |
| 7 | 1 2 1 | 13.81 | 10.02 | 245.28 | 101.23 | 92.36 | 97.02 | 20.94 | 82.54 | 75.00 | 75.00 | 18.75 | 0.00 |
| 8 | 2 2 1 | 22.04 | 9.77 | 260.96 | 92.66 | 99.63 | 121.22 | 20.90 | 91.25 | 150.00 | 75.00 | 18.75 | 0.00 |
| $F_1$ 9 | 3 2 3 | 22.24 | 10.12 | 249.76 | 89.63 | 110.23 | 159.09 | 31.70 | 84.46 | 225.00 | 75.00 | 56.25 | 10.00 |
| 10 | 3 2 1 | 22.41 | 8.10 | 250.88 | 72.56 | 113.23 | 89.00 | 42.20 | 86.33 | 225.00 | 75.00 | 18.75 | 10.00 |
| 11 | 1 1 2 | 18.08 | 10.07 | 240.8 | 95.23 | 129.63 | 121.55 | 30.79 | 94.63 | 75.00 | 37.50 | 37.50 | 10.00 |
| 12 | 2 0 2 | 17.16 | 9.67 | 232.96 | 96.63 | 112.3 | 102.73 | 37.12 | 77.64 | 150.00 | 0.00 | 37.50 | 10.00 |
| 13 | 2 3 3 | 21.57 | 11.92 | 222.88 | 101.23 | 108.56 | 146.50 | 44.48 | 99.98 | 150.00 | 150.00 | 56.25 | 10.00 |
| 14 | 0 0 0 | 13.99 | 10.94 | 230.72 | 120.3 | 96.65 | 90.24 | 44.25 | 64.53 | 0.00 | 0.00 | 0.00 | 10.00 |
| 15 | 3 3 2 | 24.41 | 9.39 | 219.52 | 103.2 | 101.23 | 124.07 | 38.09 | 84.56 | 225.00 | 150.00 | 37.50 | 10.00 |
| 16 | 3 3 3 | 20.79 | 11.09 | 221.76 | 94.56 | 99.63 | 154.01 | 45.11 | 89.63 | 225.00 | 150.00 | 56.25 | 10.00 |

| | | | | | | | | | | | | | |
|---|---|---|---|---|---|---|---|---|---|---|---|---|---|
| $F_2$ 17 | 1 2 2 | 18.24 | 11.46 | 227.36 | 92.3 | 117.56 | 124.79 | 34.52 | 90.12 | 75.00 | 75.00 | 37.50 | 20.00 |
| 18 | 3 3 1 | 23.27 | 11.41 | 231.84 | 112.25 | 124.4 | 152.33 | 45.35 | 91.32 | 225.00 | 150.00 | 18.75 | 20.00 |
| 19 | 3 2 2 | 23.12 | 11.45 | 237.44 | 105.63 | 110.23 | 220.12 | 45.26 | 93.63 | 225.00 | 75.00 | 37.50 | 20.00 |
| 20 | 0 0 0 | 17.60 | 9.29 | 231.84 | 138.25 | 101.23 | 106.92 | 44.76 | 72.63 | 0.00 | 0.00 | 0.00 | 20.00 |
| 21 | 2 3 2 | 21.01 | 14.03 | 256.48 | 94.23 | 121.36 | 89.88 | 42.56 | 95.63 | 150.00 | 150.00 | 37.50 | 20.00 |
| 22 | 2 2 2 | 22.48 | 12.14 | 315.84 | 70.26 | 125.26 | 141.61 | 36.52 | 90.32 | 150.00 | 75.00 | 37.50 | 20.00 |
| 23 | 2 1 2 | 21.98 | 10.17 | 226.24 | 97.85 | 99.63 | 129.47 | 42.87 | 90.22 | 150.00 | 37.50 | 37.50 | 20.00 |
| 24 | 2 2 0 | 22.40 | 9.47 | 263.2 | 87.56 | 114.76 | 117.77 | 33.09 | 52.38 | 150.00 | 75.00 | 0.00 | 20.00 |
