# Supplementary material for: Inductive cum targeted yield model-based integrated fertilizer prescription for sweet corn (Zea mays L. Saccharata) on Alfisols of Southern India
Source: PLoS One. 2024 Aug 26;19(8):e0307168. doi: 10.1371/journal.pone.0307168 (PMC11346652; doi:10.1371/journal.pone.0307168)
Supplement: S2 Table — (PDF) [file pone.0307168.s004.pdf]

S2 Table: Details of treatment structure, yield, uptake and initial soil analytical data for sweet corn during in strip-II

Sl No	Treatment	Yield		Initial soil available N-P-K status			Total uptake of N-P-K by crop			Fertilizer N-P-K applied			FYM applied
		(t ha^{-1})		(kg ha^{-1})			(kg ha^{-1})			(kg ha^{-1})			(t ha^{-1})
		Cob yield	Straw yield	N	P_2O_5	K_2O	N	P_2O_5	K_2O	N	P_2O_5	K_2O	
F_1 25	2 2 3	22.12	8.82	187.04	102.3	116.8	86.31	40.23	101.32	150.00	75.00	56.25	10.00
26	0 0 0	18.12	10.25	212.8	123.96	106.63	120.57	30.23	75.63	0.00	0.00	0.00	10.00
27	0 2 2	19.04	11.38	211.68	106.2	126.36	130.05	49.56	106.58	0.00	75.00	37.50	10.00
28	2 1 1	23.69	9.19	201.6	99.63	142.54	113.65	52.36	95.52	150.00	37.50	18.75	10.00
29	2 2 1	23.02	9.49	208.32	102.23	119.27	88.58	56.36	96.36	150.00	75.00	18.75	10.00
30	1 2 1	17.06	9.39	218.4	106.23	128.56	93.81	58.56	102.32	75.00	75.00	18.75	10.00
31	3 1 1	21.61	10.15	198.24	96.32	132.56	113.54	54.55	100.74	225.00	37.50	18.75	10.00
32	1 1 1	17.30	9.53	245.28	114.25	135.63	83.98	51.26	96.32	75.00	37.50	18.75	10.00
F_0 33	2 2 0	18.62	8.36	210.56	80.36	115.28	76.11	33.63	84.57	150.00	75.00	0.00	0.00
34	3 3 1	21.64	7.22	210.56	99.56	101.96	76.69	25.63	99.63	225.00	150.00	18.75	0.00
35	2 2 2	19.83	9.99	211.68	85.63	100.23	130.65	35.63	109.97	150.00	75.00	37.50	0.00
36	1 2 2	15.13	11.93	204.96	80.25	100.23	65.97	20.36	84.56	75.00	75.00	37.50	0.00
37	3 2 2	22.77	13.10	204.96	95.63	124.56	203.83	25.36	94.56	225.00	75.00	37.50	0
38	2 3 2	18.90	11.02	222.88	92.12	112.36	88.90	30.36	96.36	150.00	150.00	37.50	0
39	2 1 2	21.07	11.09	202.72	94.56	102.36	126.40	37.56	112.36	150.00	37.50	37.50	0.00

40	0 0 0	14.19	11.16	275.56	110.23	98.56	85.57	17.00	52.30	0.00	0.00	0.00	0.00
F_2 41	3 3 2	25.60	11.70	198.24	117.56	184.32	120.23	40.23	146.23	225.00	150.00	37.50	20.00
42	0 0 0	17.02	11.29	209.44	110.23	123.56	110.23	57.65	101.20	0.00	0.00	0.00	20.00
43	2 3 3	23.06	9.91	217.28	115.58	205.32	136.36	48.56	175.23	150.00	150.00	56.25	20.00
44	1 1 2	19.40	9.10	212.8	97.56	193.44	78.14	53.63	152.56	75.00	37.50	37.50	20.00
45	3 3 3	24.23	12.69	202.72	146.23	240.96	219.76	32.23	154.60	225.00	150.00	56.25	20.00
46	3 2 3	24.72	11.80	213.92	105.63	194.04	179.04	45.23	143.56	225.00	75.00	56.25	20.00
47	2 0 2	22.00	9.89	216.16	121.45	139.32	106.57	48.25	122.56	150.00	0.00	37.50	20.00
48	3 2 1	25.26	11.82	203.84	119.63	179.63	169.19	35.16	112.30	225.00	75.00	18.75	20.00
