# Supplementary material for: Inductive cum targeted yield model-based integrated fertilizer prescription for sweet corn (Zea mays L. Saccharata) on Alfisols of Southern India
Source: PLoS One. 2024 Aug 26;19(8):e0307168. doi: 10.1371/journal.pone.0307168 (PMC11346652; doi:10.1371/journal.pone.0307168)
Supplement: S3 Table — (PDF) [file pone.0307168.s005.pdf]

S3 Table: Details of treatment structure, yield, uptake and initial soil analytical data for sweet corn during in strip-III

Sl No.	Treatment	Yield (t ha^{-1})		Initial soil available N-P-K status (kg ha^{-1})			Total uptake of N-P-K by crop (kg ha^{-1})			Fertilizer N-P-K applied (kg ha^{-1})			FYM applied (t ha^{-1})
		Cob yield	Straw yield	N	P_2O_5	K_2O	N	P_2O_5	K_2O	N	P_2O_5	K_2O	
F1 49	2 2 2	21.11	10.04	257.6	108.56	199.84	132.56	45.50	111.03	150.00	75.00	37.50	10.00
50	0 0 0	17.95	10.02	263.2	103	222.04	107.52	48.56	90.10	0.00	0.00	0.00	10.00
51	3 2 2	24.24	9.76	250.88	110.23	225.28	115.59	54.89	133.63	225.00	75.00	37.50	10.00
52	2 2 0	21.87	10.44	256.48	95.63	193.01	113.06	51.25	149.66	150.00	75.00	0.00	10.00
53	2 3 2	21.46	12.16	263.2	140.26	202.72	137.84	40.23	152.33	150.00	150.00	37.50	10.00
54	3 3 1	24.10	9.69	264.32	111.26	130.12	120.46	55.56	140.97	225.00	150.00	18.75	10.00
55	2 1 2	23.48	10.27	277.76	105	160.24	145.23	58.69	121.98	150.00	37.50	37.50	10.00
56	1 2 2	19.47	10.20	265.44	99.63	199.96	133.63	54.56	144.69	75.00	75.00	37.50	10.00
F2 57	3 1 1	27.16	7.98	281.12	110.5	189.63	153.63	58.87	136.41	225.00	37.50	18.75	20.00
58	2 2 3	23.18	9.10	268.8	115.26	157.44	138.56	59.58	131.25	150.00	75.00	56.25	20.00
59	1 1 1	19.06	11.31	216.16	117.52	196.32	126.77	49.63	132.50	75.00	37.50	18.75	20.00
60	0 2 2	18.81	11.45	272.16	126.3	156.63	138.29	38.56	112.30	0.00	75.00	37.50	20.00
61	0 0 0	18.22	11.46	206.08	159.63	163.25	125.41	59.63	105.63	0.00	0.00	0.00	20.00
62	1 2 1	20.51	12.13	250.88	118.56	140.23	125.03	50.26	102.36	75.00	75.00	18.75	20.00
63	2 2 1	18.92	13.91	275.52	112.2	168.56	190.16	51.58	109.63	150.00	75.00	18.75	20.00
64	2 1 1	23.12	11.17	262.08	86.3	148.56	165.23	46.00	119.63	150.00	37.50	18.75	20.00

F0<br>65	0 0 0	14.85	11.68	285.56	100	102.23	95.63	25.26	58.60	0.00	0.00	0.00	0.00
66	1 1 2	18.13	11.75	243.04	96.63	130.23	134.09	47.56	99.65	75.00	37.50	37.50	0.00
67	3 2 3	19.53	9.87	273.28	86.36	122.25	118.27	59.63	102.25	225.00	75.00	56.25	0.00
68	2 3 3	20.13	10.66	265.44	92.56	121.56	127.49	74.26	131.23	150.00	150.00	56.25	0.00
69	3 2 1	23.47	12.12	263.2	91.23	156.96	148.56	73.50	112.25	225.00	75.00	18.75	0.00
70	3 3 3	19.93	13.24	264.32	89.63	140.3	137.50	34.56	125.63	225.00	150.00	56.25	0.00
71	3 3 2	21.97	11.57	267.68	96.36	132.63	164.51	85.03	118.56	225.00	150.00	37.50	0.00
72	2 0 2	20.60	11.69	249.76	97.56	115.26	162.53	56.56	128.36	150.00	0.00	37.50	0.00
